# Electrophysiological correlates of sustained conscious perception

**DOI:** 10.1038/s41598-024-61281-2

**Published:** 2024-05-08

**Authors:** Annika Hense, Antje Peters, Maximilian Bruchmann, Torge Dellert, Thomas Straube

**Affiliations:** 1 Institute of Medical Psychology and Systems Neuroscience, University of Münster, Von-Esmarch-Str. 52, 48149 Münster, Germany; 2 Otto Creutzfeldt Center for Cognitive and Behavioral Neuroscience, University of Münster, Fliednerstr. 21, 48149 Münster, Germany

**Keywords:** NCC, EEG/ERP, Visual awareness, Consciousness, Sustained awareness, Consciousness, Visual system

## Abstract

Previous research on the neural correlates of consciousness (NCC) in visual perception revealed an early event-related potential (ERP), the visual awareness negativity (VAN), to be associated with stimulus awareness. However, due to the use of brief stimulus presentations in previous studies, it remains unclear whether awareness-related negativities represent a transient onset-related response or correspond to the duration of a conscious percept. Studies are required that allow prolonged stimulus presentation under aware and unaware conditions. The present ERP study aimed to tackle this challenge by using a novel stimulation design. Male and female human participants (*n* = 62) performed a visual task while task-irrelevant line stimuli were presented in the background for either 500 or 1000 ms. The line stimuli sometimes contained a face, which needed so-called visual one-shot learning to be seen. Half of the participants were informed about the presence of the face, resulting in faces being perceived by the informed but not by the uninformed participants. Comparing ERPs between the informed and uninformed group revealed an enhanced negativity over occipitotemporal electrodes that persisted for the entire duration of stimulus presentation. Our results suggest that sustained visual awareness negativities (SVAN) are associated with the duration of stimulus presentation.

## Introduction

Electroencephalography (EEG) studies investigating the neural correlates of visual awareness identified a transient enhanced negative event-related potential (ERP) in response to consciously seen versus unseen stimuli around 200–300 ms after stimulus onset at occipitotemporal sites—the so-called “visual awareness negativity” (VAN; Refs.^1,2^). This negative difference wave is claimed to be the earliest and most reliable ERP signature of stimulus awareness^1^ and is in accordance with consciousness theories, which posit a central role of early sensory areas in providing conscious perception of a stimulus^3–6^. Critically, no-report paradigms have recently attempted to dissociate the proper neural correlates of consciousness (NCC) from confounded post-perceptual processes like decision-making and report^7,8^ and showed that the VAN remains under conditions in which stimuli are task-irrelevant but consciously seen^9,10^. For example, previous inattentional blindness (IB) studies using delayed unheralded awareness reports demonstrated that consciously perceived face-like stimuli reliably elicited a VAN, while later components such as the P3b depend on task-relevance of stimuli^9–13^.

While pivotal progress has been made in finding the proper NCC, previous IB studies have used relatively brief stimulus durations that did not exceed 300 ms (e.g., Refs.^9,12,13^). Other experimental blinding methods for suppressing the phenomenal awareness of visual stimuli even require durations of less than 100 ms^14–16^. The major reason for a lack of studies using prolonged stimulus durations in a contrastive design is that brief stimulus presentations are required in order to render the critical stimulus invisible for a sufficient amount of trials or participants. However, in these designs, neural activity related to maintaining a conscious percept throughout stimulus presence is hardly dissociable from transient onset- or offset-related activity. Moreover, transient activity recorded after the offset of critical stimuli might be related to interactions with neural responses to subsequent visual input. Thus, the constraint of experimental work and resulting conclusions to the use of brief stimulus durations is a strong limitation in previous NCC research. Although previous intracranial recordings in epilepsy patients provided insightful indications on sustained visual representations in occipitotemporal regions for supraliminal stimuli^17–19^, dissociating awareness-related from awareness-unrelated activity in a contrastive design that uses prolonged stimuli remains, to our knowledge, an untackled challenge.

This is particularly relevant as real-life subjective experience does not consist of short glimpses of onsets or changes of visual scenes but rather comprises sustained periods of conscious perception of unaltered visual input. It is, therefore, crucial to ask how a sustained conscious percept is encoded in neural activity. In particular, it remains unclear whether awareness-related negativities correlate with the duration of perception or whether they represent transient early responses.

To answer this question, the present study used a novel experimental design, which allowed the presentation of stimuli for longer durations in an IB paradigm involving aware and unaware participants. For this purpose, we took advantage of a phenomenon called visual one-shot learning^20^ and developed a suitable face stimulus, which is, even during repeated prolonged presentation, perceived as a random pattern by uninformed participants but easily seen as a face by informed participants. A similar effect can be observed for two-tone Mooney Faces^21^, in which a face is difficult to perceive unless when seeing a clear version of the image^22^. This phenomenon is also referred to as hidden figures, of which the most famous examples are probably the picture of a cow^23^ or the Dalmatian dog^24^. Along with stimuli containing only random lines, these faces were presented in the background of a face-unrelated visual detection task for either 500 or 1000 ms. In combination with unheralded delayed awareness ratings, the present design aimed to evaluate the modulation of the VAN by different stimulus durations while controlling for task-relevance effects and associated ERPs, such as the P3b^9,10,12,13,25^.

## Materials and methods

### Participants

86 participants were recruited at the University of Münster via public advertisements. All had normal or corrected to normal visual acuity and no history of psychiatric or neurological illness. One participant was left-handed. Participants gave written informed consent and received 10 euros per hour for participation and an additional performance–dependent bonus of up to five euros. For the final sample, only participants with sufficient behavioral and EEG data were included. In the informed group, four participants were excluded because they reported no perception of the face during the experiment. Two informed and five uninformed participants had to be excluded due to repeated perception of additional coherent patterns in scramble stimuli. Ten participants were excluded due to a high number of rejected trials in their EEG data (> 50%) and excessive skin conductance drifts, probably due to unfavorably humid environmental conditions during recording. Another three participants lacked EEG or behavioral data due to technical problems. This resulted in a final sample of 62 participants (44 female) with a mean age of 24.42 years (*SD* = 4.50) and a range of 18–39 years. The final sample consisted of 32 aware and 30 unaware participants. The study was approved by the local ethics committee.

### Apparatus

The experiment was run with Matlab (Version R2019b; Mathworks Inc., Natick, MA; http://www.mathworks.com), the Psychophysics Toolbox^26,27^, and the Eyelink Toolbox^28^. An iiyama HM903DT monitor at 60 Hz with a resolution of 1920 × 1024 pixels was used for stimulus display. The viewing distance amounted to 60 cm. Participants pressed the space bar of a standard keyboard to respond to targets during the visual distractor task. A chin rest was used to prevent head movements during the experiment.

### Experimental design and statistical analysis

#### Stimuli and presentation

**Figure 1:**
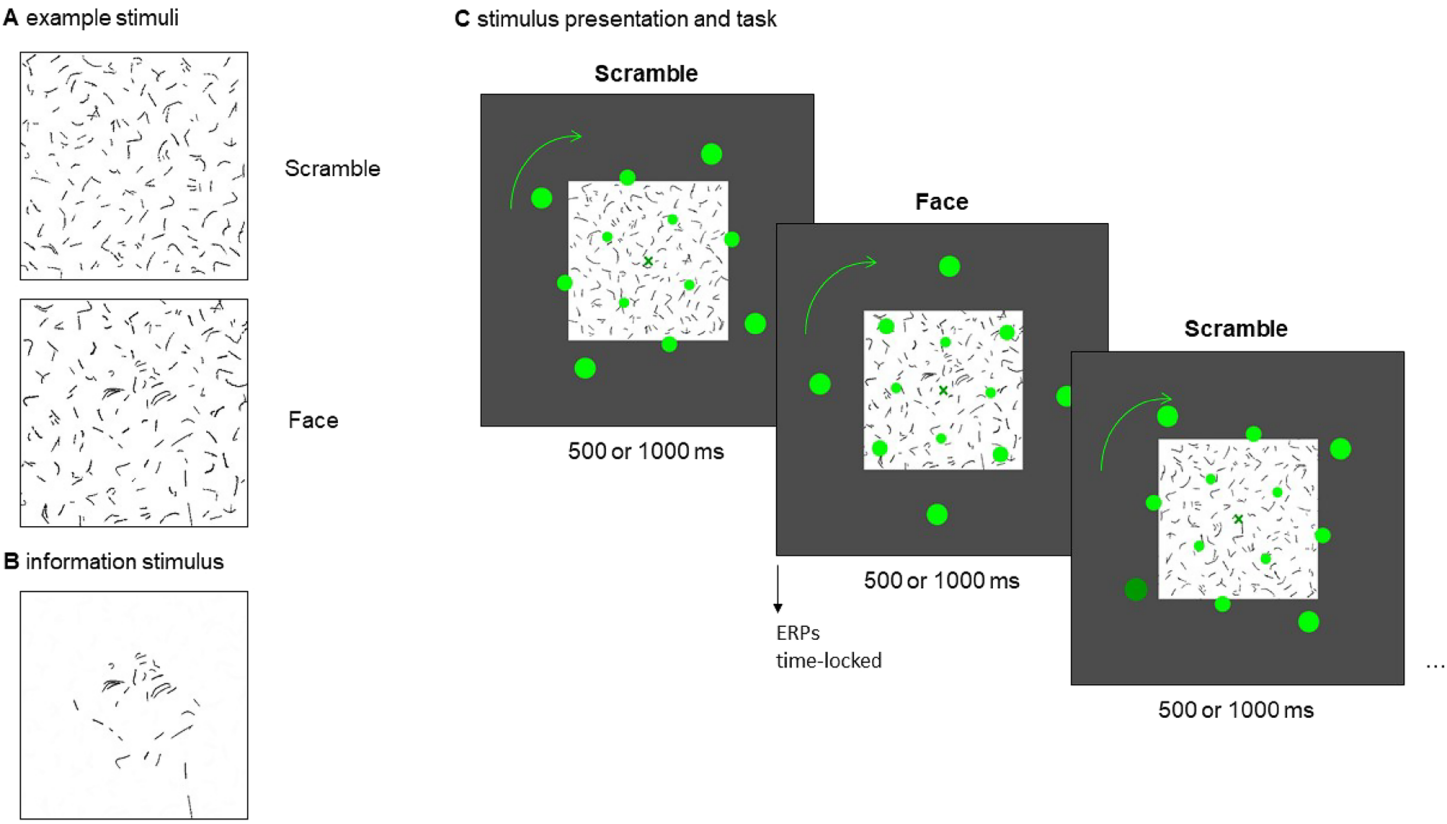
(**A**) Example of a scramble stimulus and the face stimulus used in the main experiment. (**B**) Stimulus used during the information phase in which lines not directly pertaining to the face itself were faded out. (**C**) Stimulus presentation during the main experiment with continuous visual distractor task in the foreground. Background stimuli were presented for either 500 or 1000 ms. Participants were instructed to press the space bar whenever they detected a brief lumination change in one of the green dots (last display). ERPs were time-locked to the onset of the stimuli in the background.

The experimental design was based and adapted on similar IB studies with shorter presentation of critical stimuli embedded in meaningless line stimuli^9,10,12,13,29^. However, the facial stimulus used in the current study was designed based on a single line drawing of a human face from Google Images with its view oriented obliquely upwards to the right and modified to fit the present experimental paradigm. Specifically, the lines of the original image were fragmented into short segments. Additional similar lines were arranged around the face (Fig. 1). Based on pilot experiments, the face stimulus was designed to depend on so-called visual one-shot learning^20^, which is a rapid and long-lasting insight on how to see the face in the picture once being informed about it by exposure to a less ambiguous version of the same image. The face is then recognized by informed participants, while uninformed subjects see nothing but meaningless random lines, even if the face is presented for longer durations compared to typical consciousness experiments. Additionally, 12 line stimuli without a face configuration were generated. Lines of the face stimulus were rearranged in three different ways so that only meaningless line patterns resulted. Each of these 3 stimuli was rotated by 90, 180, and 270 degrees, leading to 12 different scramble stimuli.

The background of the stimuli was white and the lines were black. The proportion of black pixels was kept constant at 30% for each stimulus. For the face stimulus and an example scramble stimulus see Fig. 1A. Using 13 different stimuli (1 face stimulus, 12 scramble stimuli), each line configuration could appear with identical probability while simultaneously providing sufficient variability in background stimuli. Stimuli consecutively appeared centrally on a gray background in a size of 300 × 300 pixels with a duration of either 500 or 1000 ms. The 500 ms duration condition firstly aimed to replicate VAN findings of previous IB studies^12,13^ using the newly designed face stimulus. At the same time, introducing a slightly longer stimulus duration allows precluding awareness-related neural activity occurring within 500 ms after the stimulus to be related to altered processing of subsequent visual input. It is referred to as the *short* stimulus condition. The 1000 ms duration condition then served to specifically address the present question on the modulation of the awareness-related negativity by a prolonged stimulus duration and associated conscious representation. It is therefore referred to as the *long* stimulus condition. For both the short and long stimulus condition, differential responses to face versus scramble are expected for the aware but not for the unaware group.

Each of the 13 stimuli was presented 100 times per duration, resulting in a total of 2600 stimulus presentations (referred to as trials). Stimulus order was pseudorandomized by concatenating randomized sequences of the 13 different stimuli on the condition of no repetition of the same stimulus. Simultaneously to the presentation of the line stimuli, 12 green dots were presented in the foreground on 3 circular paths (4 dots on each circle) with a radius of 2.4, 4.5, and 6.5° visual angle, respectively (for a stationary image of the dots, see Fig. 1C). The dots, with radii of 0.32, 0.41, and 0.52°, rotated with a constant angular velocity of 1.48 radians/s around the fixation cross. In 10 percent of randomly distributed trials, but with a minimal gap of 4 trials, a randomly chosen dot slightly decreased in luminance for 200 ms (from (0, 255, 0) to (0, 127.5, 0) in RGB). The rotation direction changed every 30 trials on average (jitter + / − 10 trials) from clockwise to counterclockwise and vice versa. At all times, a green fixation cross was presented in the center of the screen ((0, 153, 0) in RGB). Whenever the participants pressed the space bar, the fixation cross changed its color to either brighter green (indicating a hit, i.e. a button press within 2 s after the target, (77, 255, 77) in RGB) or red (indicating a false alarm, (255, 0, 0) in RGB).

#### Procedure

The stimulus presentation was physically identical to all participants for the main experiment and a preceding practice session. All participants were instructed to press the space bar whenever they detected a luminance decrease in one of the dots, to ignore the content of the background line stimuli, and to avoid eye movements and blinks during the experiment (except for the breaks).

Before starting the main experiment, participants were either informed or left uninformed about the presence of a face in one of the stimuli. During the instruction for informed participants, the face stimulus was presented five times for 5000 ms during which the face was accentuated by fading out all lines that did not pertain to the face itself (see Fig. 1B). Participants were then asked to report whether they detected the face. In the case of affirmation, the face stimulus was presented five times without accentuation, again followed by the question of whether they still detected the face. In the case of negation, the instruction phase was repeated but not more than three times. If participants had not affirmed both questions within these three repetitions, the experiment was aborted. Yet, this case did not occur.

This procedure was chosen to ensure that informed participants knew about and were able to see the face even when instructed to pay attention to the visual distractor task. Uninformed participants were only told about random black lines in the background. To familiarize participants with the experimental task, all performed a practice session with decreased task difficulty (the luminance change of dot targets was slightly higher than in the main experiment; from (0, 255, 0) to (0, 51, 0) in RGB). The main experiment then comprised 4 blocks with 650 trials each, separated by breaks to relax the eyes and uphold concentration. By pressing a button on the keyboard, participants were able to continue the experiment. Feedback in the form of written statements in 5 grades according to participants’ performances was presented after each block. Color changes of the fixation cross (cf. section “Stimuli and presentation”) further served to uphold motivation and attention to the dot task. Each block lasted around 8 min, resulting in a total experiment duration of 33 min. The final performance-dependent monetary bonus was calculated based on the rates of hits (H), misses (M) and absolute amount of false alarms (F), using the formula $B= \left(H-0.25*M\right)*500-F*\left(\frac{500}{T}\right)$, where B refers to the total bonus and 500 refers to the maximal bonus of 500 cent. T is the total amount of targets. The maximal bonus of 5 euros was achieved by a hit rate of 1 and 0 false alarms, while the minimal accessible bonus was 0 euros.

Immediately after the experiment, participants completed an unannounced awareness assessment to ensure sufficient sensitivity to identify all participants who detected at least some visual stimuli. They were first openly asked whether they noticed anything in the background and afterwards, whether or not they perceived the face in the background. Participants were additionally asked if they had perceived any other imaginary coherent figures to exclude unintended perceptions of other arbitrary figures in the stimuli. To prevent misclassification of aware participants to the unaware group by underestimation of awareness, e.g., from relying on the last block of their estimate, we additionally asked when they first noticed a face (block 1, 2, 3 or 4), how clearly they had seen the faces on a scale from 1 to 10 and how many faces they had seen (the frequency rating was asked to be indicated per block for facilitation).

#### Behavioral data analysis

Task performance was quantified by *d*’^30^ and median response times (RTs) for each participant, and analyzed in JASP (JASP Team (2019). JASP (Version 0.11.1)). Eight participants showed extreme values for either the hit rate or false alarm rate (hit rate = 1 or false alarm rate = 0) in which cases *d*’ is indeterminable. We, therefore, applied the log-linear approach by adding 0.5 to both the number of hits and the number of false alarms and adding 1 to the number of signal trials and the number of noise trials before calculating the hit and false alarm rates^31^. Due to the continuous stimulus presentation in the dot task, false alarms were examined relative to the number of nontarget time intervals of the same length as the 2s response window for hits^32^. Median RTs were used to summarize single-trial RTs in order to be more robust against skewness and outliers. In order to investigate whether neural differences in the critical awareness contrast could also be ascribed to differences in attentional allocation, *d*’ and median RTs were compared between aware and unaware subjects using an independent-sample two-sided t-test. As some of our conclusions rely on null effects, we report both frequentist and Bayesian inference and effect sizes^33^. T-tests were conducted using the conventional a priori threshold of α < 0.05. Bayes factors (BFs) quantify how much more likely one hypothesis is compared with another, with BF_01_ denoting the evidence for the null hypothesis (absence of an effect) and BF_10_ the evidence for the alternative hypothesis. BFs are interpreted based on the conventions by Jeffreys^34^. Effect sizes are reported as Cohen’s *d*.

#### Eye-tracking

To ensure that participants were constantly looking at the fixation cross, we used an eye-tracker to continuously track and evaluate the gaze position during the experiment, stopping experimental presentation whenever the gaze deviated more than 150 pixels in any direction from the fixation cross. Aborted trials were added to the end of the block. Eye-tracking was measured with the Eyelink 1000 eye-tracker (SR Research Ltd., Mississauga, Canada). Participants were asked to place their heads on a chin rest. The right eye was recorded with a sampling rate of 1000 Hz. Before the experiment, the eye-tracker was calibrated using a five-point calibration procedure and automatically initiated again whenever gaze deviation was detected in 5 consecutive trials. For seven participants, eye-tracking data were not recorded due to technical difficulties. Eye-tracking was used exclusively for online gaze control. We chose not to perform any secondary offline analyses as the experiment’s design discouraged eye movements at all times.

#### EEG recording and preprocessing

A 64-channel BioSemi active electrode system (BioSemi B.V., Amsterdam, Netherlands) was employed to collect electrophysiological data. Electrodes were placed using the equiradial system conforming with BioSemi electrode caps. Furthermore, vertical and horizontal eye movements were recorded with two electrodes attached above and below the left eye (VEOG) and two electrodes attached to the right and left outer canthi (HEOG). Instead of ground and reference, the BioSemi EEG system uses a CMS/DRL feedback loop with two additional electrodes. Electrical potentials were recorded with a sampling rate of 512 Hz, and electrode offsets were held below 20 µV. A built-in analog anti-aliasing low-pass filter of 104 Hz was applied prior to digitization.

EEG data was preprocessed using Brain Electrical Source Analysis (BESA) Research 6.0 (BESA GmbH, Gräfeling, Germany). Offline data were re-referenced to average reference and filtered with a high-pass forward filter of 0.2 (6 dB/oct) and a 30 Hz low-pass zero-phase filter (24 dB/oct). Line noise was removed by applying a 50 Hz notch filter. Eye movements were corrected using the automatic eye-artifact correction method implemented in BESA^35^. Further artifacts were removed using the semi-automatic, PCA-based artifact topography algorithm implemented in BESA by manually selecting artifacts. All corresponding components were removed automatically if they explained more than 80% variance of the artifact topography. The continuous signal was segmented into epochs of 500 ms before to 1200 ms after the onset of the critical background stimuli. Trials were baseline-corrected based on the average of the prestimulus interval from  − 500 to 0 ms, which was chosen to protect against the influence of previous stimuli due to the absence of interstimulus intervals. Trials containing muscle artifacts, electrode jumps, or amplitudes exceeding a threshold of 100 uV were rejected based on visual inspection, and bad channels were interpolated. In the EEG data of the final sample of 62 participants, an average of 2094.6 trials (*SD* = 107.76) were included in statistical analysis, and 3.61 electrodes (*SD* = 1.75) were interpolated with no differences between groups (included trials: *t*(60) = 0.16, *p* = 0.876; interpolated channels: *t*(60) = − 0.96, *p* = 0.341).

#### EEG data analysis

For statistical analysis, we only included trials that were not aborted due to excessive eye movements according to the eye-tracker. Moreover, trials containing a dot target were excluded from further analysis. To attain adequate comparability of the ERP baselines, we only included scramble trials with no face stimulus preceding them. The 12 different scramble stimuli were averaged into a single scramble condition. Differential responses to face versus scramble stimuli were computed within each group and duration condition, and used for awareness contrasts.

To choose the most sensitive electrodes for the question of a sustained awareness effect on the VAN, we first identified the electrode with the largest early VAN across conditions in the typical time window of 200–300 ms according to previous IB studies that used similar line stimuli^9,12,13^. All electrodes posterior to midline were compared according to their effect sizes of group differences across both short and long condition for the mentioned time window, quantified by Cohen’s *d.* The electrode showing the largest effect and its immediate neighbor electrodes were selected for further analysis (see also Ref.^9^). The strongest negative signal was observed in P7 (*M* = − 0.45 µV, *t*(60) = − 2.928, *d* = 0.74). Averaged signals of electrode P7 and its immediate lateral neighbors PO7 and P9 were chosen for further analysis, with particular interest in a late time window (500 to 1000 ms), which did not overlap with the early VAN effect (see the following paragraph for more details).

The within-subject comparison of face versus scramble for each condition (aware short, unaware short, aware long, unaware long) as well as between-subjects awareness contrasts for both the short and long condition were then computed in two different intervals of interest, an early and a later time window. An early time window (200–500 ms) comprised latencies of awareness-related negativities found in previous IB studies^12,13^ but accounted for the extended stimulus duration. The late time window was defined as 500–1000 ms. For the early time window, a visual awareness-related negativity was expected to occur for both the short and long stimulus condition. ERP responses to the long stimulus condition that occur after 500 ms, in the late time window, should be related to awareness of the face stimulus. In the short but not in the long stimulus condition, ERPs after 500 ms are influenced by possible offset responses or altered processing of subsequent stimuli due to previous face perception. For this reason, the interpretability of neural responses occurring in the late time window in the short condition is limited, which is also true for other NCC studies using brief stimulus durations. Differential ERP waves (face–scramble) for each condition were averaged across the electrodes of interest and the respective time windows. The a priori defined within- and between-subject contrasts were calculated using frequentist and Bayesian inference. We used unidirectional one-sample (for within-subject contrasts) and independent-sample t-tests (for awareness contrasts) with a conventional a priori threshold of *α* < 0.05 and report Bayes Factor as well as effect sizes (Cohen’s *d*). Besides the focus on the temporal dynamics of the awareness-related negativities, we also evaluated whether an awareness-related P3b would be found in the long condition. While the P3b has often been assumed to be strongly correlated with conscious awareness^36^, this ERP component was not related to stimulus awareness in IB studies when stimuli were task-irrelevant^12,13,29^. Following previous IB studies, we investigated the P3b comparing aware versus unaware participants in the time interval from 400 to 600 ms at electrode Pz^12,13^. Only the long stimulus condition allows to exclude the aforementioned confoundation with offset-related neural responses in the investigated time window.

## Results

### Behavioral data

#### Awareness ratings

In an unheralded posthoc awareness assessment, the 32 aware participants of the final sample reported that they had seen the face 49.22 times on average (*SD* = 47.56) during the experiment, with a minimum of nine and a maximum indication of 200 times. Three participants reported that they first noticed the face in block 2, another participant in block 3, and the rest of the aware participants reported that they first perceived the face in the first block. The clarity of the face was rated with an average of 8.44 (*SD* = 2.58) out of ten. However, note that the reliability of these awareness ratings is unclear.

#### Task performance

**Figure 2:**
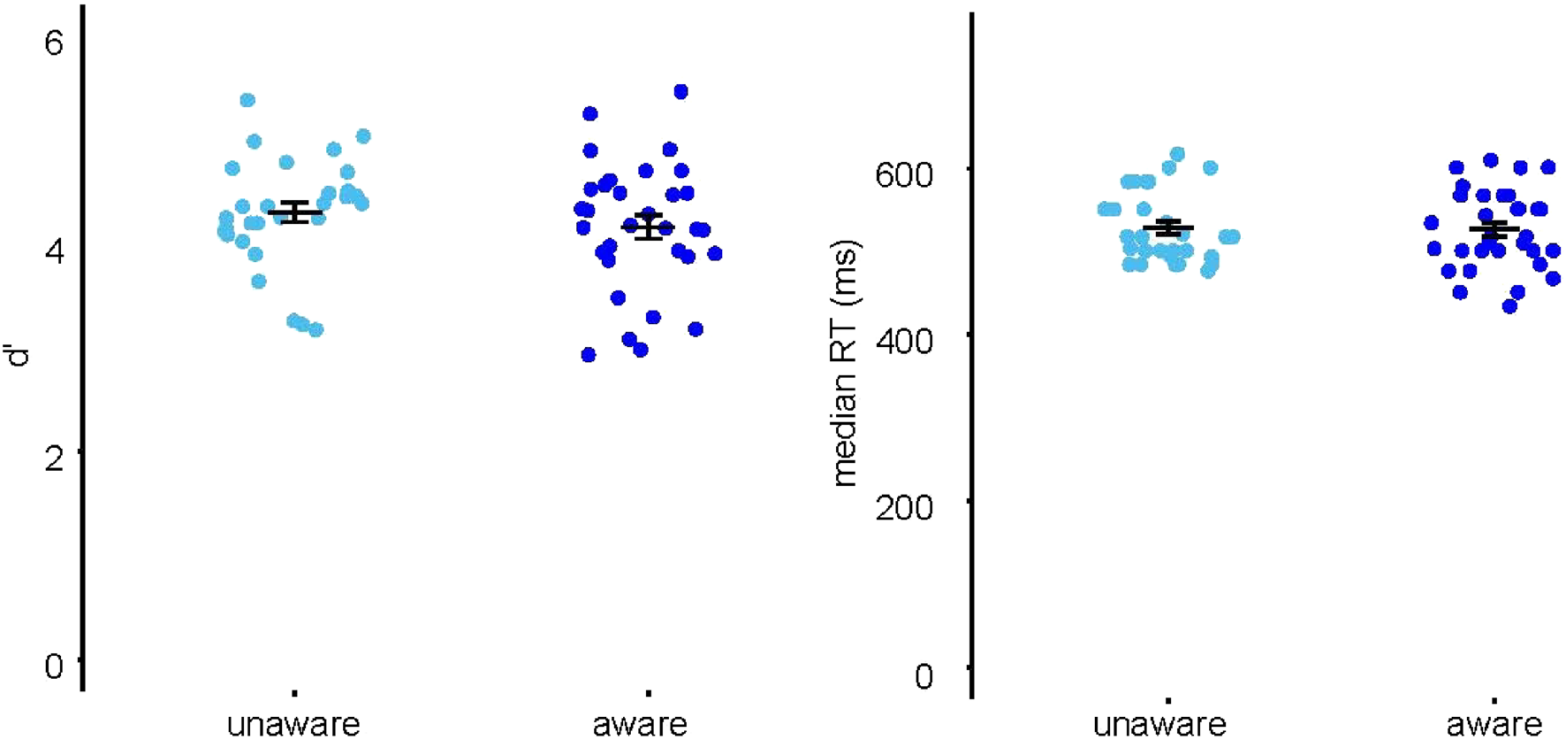
Behavioral results. Performance in the visual distractor dot task, quantified by *d*’ and median RTs in ms. Error bars present SEs.

 In order to investigate whether neural differences between the included 32 aware and 30 unaware participants could also be due to differences in attentional allocation to the visual distractor task, task performance in the visual distractor task, quantified by *d*’ and median reaction times (RT), were compared between these groups. An illustration of the experimental design is presented in Fig. 1C. Levene’s test of equality of variances indicated no violation of the assumption of homoscedasticity for loglinear corrected *d*’ (*F*(1) = 1.26, *p* = 0.267) as well as for median RTs (*F*(1) = 0.68, *p* = 0.411). Log-linear corrected *d*’ did not show significant differences between groups (*t*(60) = 0.97, *p* = 0.335, *BF*_01_ = 2.60; Fig. 2). Median reaction times did not show significant group differences either (*t*(60) = 0.15, *p* = 0.884, *BF*_01_ = 3.83; Fig. 2).

#### Electrophysiological measures of awareness

**Figure 3:**
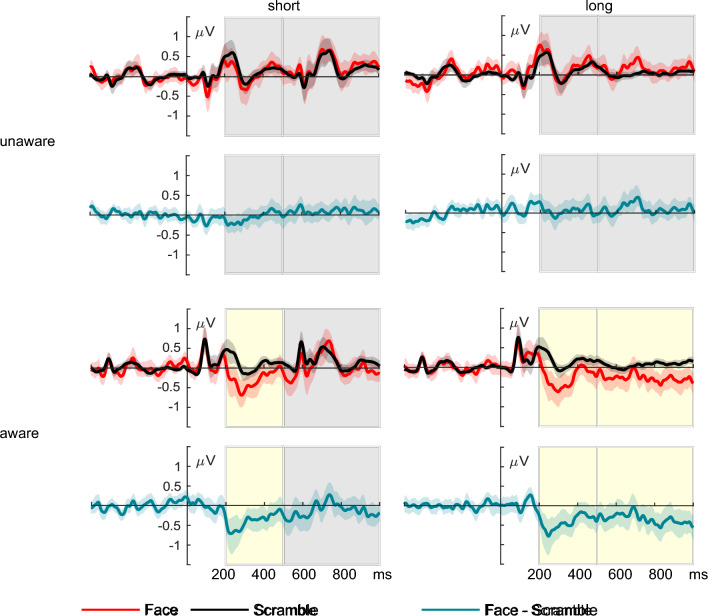
Averaged ERPs in the electrodes of interest (P9, PO7, P7) separated by each group and stimulus duration. Difference waves (face–scramble) are plotted below, respectively. Intervals of interest marked by gray boxes were used for ERP average contrasts. Yellow boxes mark significant ERP differences across the respective time intervals. The shaded area around ERP waveforms depicts the 95%-bootstrap confidence interval.

Grand-averaged ERPs across the selected electrode cluster for faces and scrambles are displayed in Fig. 3, separated by group and duration conditions, with difference waves presented below. In the early time window, ERPs elicited by the face stimulus were more negative than ERPs elicited by the scramble stimuli in the aware group in both the short and long stimulus condition (short: *t*(31) =  − 3.11, *p* = 0.002, *d* =  − 0.55, *BF*_10_ = 19.18; long: *t*(31) =  − 3.37, *p* = 0.001, *d* =  − 0.60, *BF*_10_ = 34.95). No negative deflection for face versus scrambles was evident in the unaware group during the early time window, neither in the short nor the long stimulus condition (short: *t*(29) =  − 0.88, *p* = 0.191, *d* =  − 0.162, *BF*_01_ = 2.245; long: *t*(29) = 0.601, *p* = 0.725, *d* = 0.11, *BF*_01_ = 7.70). Furthermore, in the late time window, a significant negativity in response to faces versus scrambles was only observed in the long stimulus condition in the aware group (*t*(31) =  − 2.46, *p* = 0.010, *d* =  − 0.44, *BF*_10_ = 4.98), but not in the other conditions (short, aware: (*t*(31) =  − 0.98, *p* = 0.167, *d* =  − 0.17, *BF*_01_ = 2.06; short, unaware: *t*(29) = 0.91, *p* = 0.814, *d* = 0.17, *BF*_01_ = 9.05; long, unaware: *t*(29) = 1.23, *p* = 0.885, *d* = 0.22, *BF*_01_ = 10.51).

**Figure 4:**
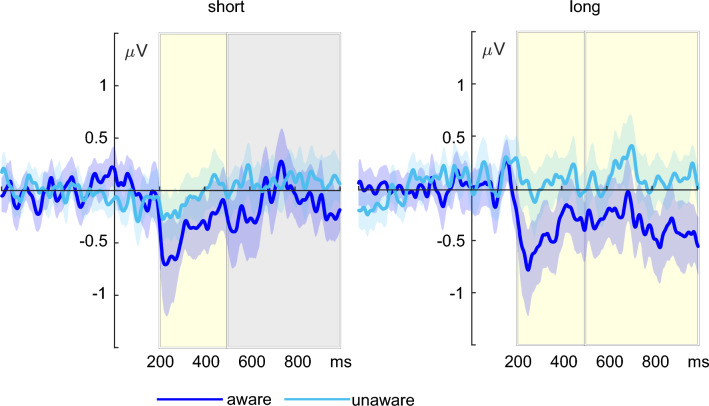
Electrophysiological results for the short condition on the left and the long condition on the right. The awareness contrast compared difference waves (face—scrambles) between aware (dark blue) and unaware (light blue) participants in the two intervals of interest, respectively. ERPs are averaged across the electrodes of interest (P9, PO7, P7). Gray boxes indicate the intervals of interest in which statistical analyses were calculated. Yellow boxes indicate time intervals showing significant differences. The shaded area around ERP waveforms depicts the 95%-bootstrap confidence interval.

**Figure 5:**
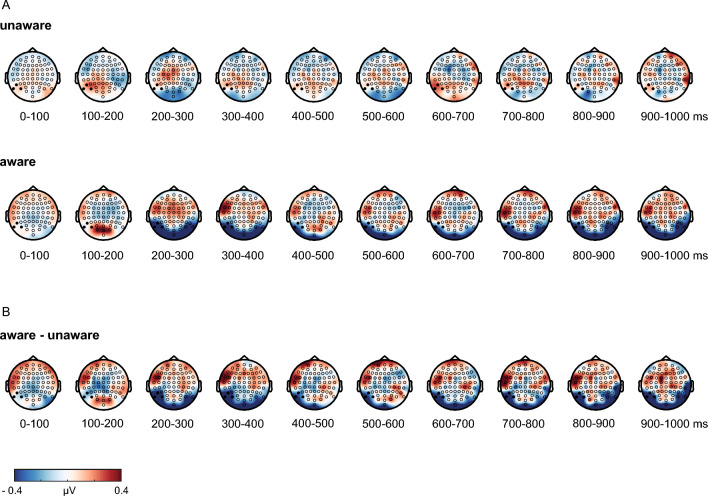
Topographical maps for face-scramble differences in the long stimulus condition. (**A**) Differential activity between long faces and scrambles is plotted in 100 ms steps in the unaware and aware group, respectively. (**B**) Difference scalp topographies of aware versus unaware participants. A sustained posterior negativity is seen on left-lateralized electrode sites beginning around 200 ms after stimulus onset.

Comparing average ERP difference waves (face versus scramble) between aware and unaware participants in the early window revealed a larger negativity for aware compared to unaware participants in the short condition (*t*(60) =  − 1.99, *p* = 0.025, *d* =  − 0.51, *BF*_*10*_ = 2.60*)* as well as in the long condition (*t*(60) = − 3.09, *p* = 0.002, *d* =  − 0.79, *BF*_*10*_ = 24.62). The awareness contrast for average ERP difference waves in the late window confirmed an additional negative deflection for aware compared to unaware participants in the long stimulus condition (*t*(60) =  − 2.70, *p* = 0.005, *d* =  − 0.69, *BF*_*10*_ = 10.03), which was not significant in the short stimulus condition (*t*(60) =  − 1.31, *p* = 0.097, *d* =  − 0.33, *BF*_*01*_ = 1.06). ERPs for the aware and unaware group are displayed in Fig. 4. Figure 5 illustrates the topography of face-scramble effects in both the aware and unaware group for the long stimulus condition, displayed in 100 ms steps.

An exploratory analysis repeated the previous analysis in the symmetric electrodes on the right (P8, PO8, P10). The findings for a left-hemispheric cluster described in the previous paragraphs were only partially observed on the right hemisphere. In the early time window, ERPs in response to faces versus scrambles were significantly more negative in the short but not in the long stimulus condition in the aware group (short: *t*(31) =  − 1.93, *p* = 0.031, *d* =  − 0.34, *BF*_10_ = 1.89; long: *t*(31) =  − 1.60, *p* = 0.06, *d* =  − 0.28, *BF*_01_ = 0.90). No negative deflection for faces versus scrambles was evident in the unaware group during the early time window, neither in the short nor the long stimulus condition (short: *t*(29) = 1.51, *p* = 0.929,* d* = 0.276, *BF*_01_ = 1.85; long: *t*(29) =  − 0.76, *p* = 0.228, *d* =  − 0.14, *BF*_01_ = 3.95). In the late time window, a significant negativity in response to faces versus scrambles was observed in the long stimulus condition in the aware group (*t*(31) =  − 2.63, *p* = 0.007, *d* =  − 0.47, *BF*_10_ = 3.52) but not in the other conditions (short, aware: (*t*(31) =  − 0.15, *p* = 0.442, *d* =  − 0.03, *BF*_01_ = 5.24; short, unaware:* t*(29) = 1.02, *p* = 0.841, *d* =  − 0.19, *BF*_01_ = 9.55; long, unaware: *t*(29) = -0.62, *p* = 0.270, *d* =  − 0.11, *BF*_01_ = 2.986). Comparing average ERP difference waves (face versus scramble) between aware and unaware participants in the electrodes P8, PO8 and P10 in the early window revealed a larger negativity for aware compared to unaware participants in the short condition (*t*(60) = − 2.42, *p* = 0.009, *d* = − 0.61, *BF*_10_ = 5.64) but not in the long condition (*t*(60) = -0.91, *p* = 0.18, *d* = − 0.23, *BF*_01_ = 1.71), with anecdotal evidence for the absence of the effect in the long condition. The awareness contrast for average ERP difference waves in the late window showed no negative difference in aware compared to unaware participants neither in the short (*t*(60) =  − 0.70, *p* = 0.243, *d* =  − 0.18, *BF*_01_ = 2.13), nor in the long stimulus condition (*t*(60) =  − 1.46, *p* = 0.075, *d* =  − 0.37, *BF*_01_ = 0.87).

Besides the focus on the temporal dynamics of the awareness-related negativities, we also evaluated whether an awareness-related P3b would be found in the long condition. Following previous IB studies, we investigated the P3b by comparing aware and unaware participants in the time interval from 400 to 600 ms at electrode Pz. The results revealed substantial evidence against a late positive potential in the long condition (*t*(60) = − 1.2, *p* = 0.885, *d* =  − 0.31, *BF*_01_ = 7.71). The topographic plot (Fig. 5) also indicates no reliable awareness-related P3b at Pz or neighboring electrodes.

## Discussion

The present study isolated awareness-related ERPs associated with the duration of a consciously perceived stimulus, using a prolonged presentation of line stimuli, among which an abstract human face was only perceived by informed participants. In an IB experiment, we detected a sustained visual awareness negativity (SVAN) at posterior electrodes during prolonged stimulus presentation. The ERP results replicate a typical early VAN at occipitotemporal electrodes starting around 200 ms after stimulus onset, which was found to persist for the duration of a consciously perceived prolonged face stimulus. Thus, beyond typically investigated time epochs, an awareness-related negativity was also significant in a late time window ranging from 500 to 1000 ms for long but not for short stimuli. The early VAN therefore appears to be associated with the duration of the perceived stimulus.

As described above, the usage of brief stimulus durations in ERP studies, which aim to evaluate awareness-related neural activity, might lead to interpretative problems for effects occurring after the offset of the stimulus. Moreover, conscious visual experience usually does not span just a brief 100 to 200 ms of the VAN time window reported in previous studies^12,13,15^. To our knowledge, the present study is the first one that provided prolonged stimulus presentations amounting to 500 and 1000 ms, while including both an aware and an unaware condition. In the case of the short stimulus condition, the presence or absence of effects in the late time window might also be due to altered processing of subsequent visual input as a consequence of previous perception of the face. However, in contrast to the short stimulus condition, the long stimulus condition allows for excluding these confounding factors both across the early and the late time window.

Considering the location of the present awareness effects at occipitotemporal sites, the later negativity may correspond to a sustained prolongation of an early VAN reported around 200–300 ms after stimulus onset in previous IB studies^12,13^. The scalp topography throughout the duration of the long stimulus condition indicated a sustained negative deflection for aware participants at left-lateralized occipitotemporal sites, which was absent in the unaware group.

We would like to note that we cannot rule out that the found sustained activation might be based on different processing stages with different overlapping components. However, the present results are in accordance with intracranial recordings that show sustained representations of prolonged supraliminal stimuli in occipitotemporal areas, in contrast to transient frontoparietal representations^17–19^. Gerber et al.^17^ showed diminishing real-time fidelity of sustained stimulus presentations along the visual hierarchy. These differing temporal dynamics are explained to be an expression of varying function roles of cortical areas^17^, which may correspond to different aspects of perception^19^. Posterior regions in visual sensory areas are surmised to contribute to maintaining an ongoing visual percept while more anterior regions would contribute to discrete aspects of perception, that is those that do not necessarily rely on stimulus presence, like perceptual updating, identification or categorization^17,19^. However, previous studies using prolonged stimulus durations lack an awareness contrast and proper control for the task relevance of stimulus (which is not necessarily related to overt response requirements). Therefore, their results cannot be unambiguously interpreted as awareness-related signals. Using a suited awareness contrast and an established IB design allows disentangling awareness-related neural activity from post-perceptual effects associated with the task relevance of a stimulus. Our results suggest that posterior awareness-related negativities may not only reflect the entrance of a visual percept into consciousness but correspond to the duration of a seen versus unseen stimulus.

Yet, sustained and transient temporal dynamics are mixed up in typical occipitotemporal activations since EEGs represent averages of thousands of distributed neurons, which might involve areas with differing temporal dynamics. For example in our study, the present differential activation to aware versus unaware faces might represent both an initial transient as well as sustained neural activity located in several areas of the face processing network.

An initial differential activation to aware versus unaware faces might represent an N170-related onset response, which has been shown to be an early stimulus-specific response for faces that also covaries with conscious perception^12,13^. The N170 usually peaks around 200 ms after stimulus onset and shows to be more consistent on the right-lateralized hemisphere^37^. Inspection of the data shows bilaterally increased N170-like responses (at about 210 ms) in all aware versus unaware conditions in our study. Also, a more transient response in right-lateralized electrodes appears to accord with the scalp topography of the long stimulus condition (Fig. 5). Thus, right-sided early activity might be a necessary condition for the structural representation of faces. VAN-like more sustained responses to faces might strongly involve the left hemisphere and the general activation of a network for face processing. Considering previous intracranial studies, the SVAN might mainly represent amplified processing of low-level stimulus features, maintained to provide a sustained conscious experience^17^. However, it is important to note that the N170 was not specifically investigated in the present study. Due to overlapping latencies of the N170 and VAN and delayed peak latencies of N170 responses to abstract faces compared to face photographs^38^, we decided not to try to separate these components in the present study. Furthermore, we used a novel and highly specific face stimulus, based on line patterns and an unusual perspective of the tilted face, which pointed to the right. These points may have affected the electrophysiological outcomes. Thus, our study can only be the starting point to investigate these questions in detail in the future. For example, future studies with other stimuli and other electrode configurations might result in other outcomes.

Our present findings may support consciousness theories assuming a pivotal role of activation in posterior visual areas for consciously experiencing and maintaining specific stimulus contents. For example, recurrent processing theory (RPT) assumes that recurrent processing in posterior areas is both necessary and sufficient for conscious experience since they enable integration of low-level and high-level features into an unitary whole^4,5^. However, to our knowledge, RPT makes no specific claims on how a conscious percept is maintained. From a RPT perspective, a sustained posterior negativity might correspond to persistent recurrent processing in sensory areas. According to an ongoing adversarial collaboration, which aims to test theoretical predictions of two prominent consciousness theories^18^, proponents of the Integrated Information Theory (IIT^6^ propose that activity is sustained for the duration of the visual experience in visual areas, while, in contrast, the Global Neuronal Workspace Theory (GNWT^39,40^ predicts a neural ignition in fronto-parietal areas at stimulus onset and offset, marking updating of the neuronal workspace. However, in accordance with previous studies^9,11–13,15,25^, we found no support for the role of the P3b in stimulus awareness, contradicting predictions of the GNWT that stress the P3b as an electrophysiological correlate of conscious ignition^36^. Previous studies show that the increased P3b amplitude for aware compared to unaware conditions is only present in task-relevant conditions and, therefore, not reflecting neural processes related to stimulus awareness per se but task-related processes like decision-making^12,13^. Nevertheless, late positivities have been argued to reflect the depth of stimulus processing and may thus contribute to the extent of conscious experiences^41^.

Our study has both strengths and limitations. The crucial advantage of the present design is the ability to isolate awareness-related neural activity from task-related and awareness-unrelated processes while using prolonged, high-contrast stimuli. On the other hand, delayed awareness reports preclude precise single-trial assessment of awareness. This might lead to underestimating NCCs. Yet, we found early awareness-related negativities, typically small effects, which underlines the sensitivity of the current design and therefore should not alter our main conclusions for modulation of the VAN by prolonged stimulus durations. Furthermore, one could argue that putatively unaware participants may have perceived but swiftly forgotten the faces (inattentional amnesia; Ref.^42^). However, it has been demonstrated that the inability to report stimuli during IB stems from perceptual rather than memory deficits^43^. Abstraction of the used face stimulus and high memorability of unexpected faces further make this explanation rather improbable. Future studies are needed that investigate how the ongoing perceptual experience is reflected in neural activity and which temporal dynamics contribute to a sustained subjective experience of our environment. Transient and sustained awareness-related responses may reflect different aspects of conscious perception. Specifically, the precise role of frontoparietal areas and related late potivities as well as the underlying mechanism of early awareness-related negativities should be the subject of future research. Notably, it is crucial to dissociate neural activity associated with conscious perception from that related to the report or task-related decision-making in order to contribute to adjudicating between existing theoretical approaches and uncover the underlying neural mechanism of consciousness.

## Conclusion

In conclusion, the present study provides, to our knowledge, the first evidence of a sustained visual awareness negativity. This SVAN was found in occipitotemporal electrodes and appeared to correspond to the duration of a consciously perceived abstract face stimulus. Our results corroborate the notion that continuous visual perception is associated with sustained activity in sensory brain areas beyond the initial onset-related activity.

## Data Availability

The datasets analysed during the current study are available from the corresponding author on reasonable request.
